# Apoptosis activation during *Lagovirus europaeus*/GI.2 infection in rabbits

**DOI:** 10.3389/fmicb.2023.1308018

**Published:** 2024-01-25

**Authors:** Dominika Bębnowska, Rafał Hrynkiewicz, Karolina Wiśniewska, Magdalena Żabińska, Estera Rintz, Karolina Pierzynowska, Paulina Niedźwiedzka-Rystwej

**Affiliations:** ^1^Institute of Biology, University of Szczecin, Szczecin, Poland; ^2^Department of Molecular Biology, Faculty of Biology, University of Gdańsk, Gdańsk, Poland

**Keywords:** *Lagovirus europaeus*, rabbit haemorrhagic disease, RHDV, apoptosis, rabbits

## Abstract

Rabbit Haemorrhagic Disease (RHD) is a severe disease caused by *Lagovirus europaeus*/GI.1 and GI.2. Immunological processes such as apoptosis are important factors involved in the pathogenesis of Rabbit Haemorrhagic Disease (RHD). The process of programmed cell death has been quite well characterized in infection with GI.1 strains, but apoptosis in infection with GI.2 strains has not been widely studied. This is particularly important as several studies have shown that significant differences in the host immune response are observed during infection with different strains of *Lagovirus europaeus*. In this study, we analyzed the gene expression, protein levels and activity of key apoptotic cell death factors in the spleen, kidney, lung, and heart of rabbits. As a result, we showed that there is a significant increase in *caspase-3, Bax, Bcl2* and *Bax/Bcl2* mRNA gene expression ratio in organs of infected animals. Our results show also increased levels of cleaved caspase-3, caspase-6 and PARP. Moreover, significant activity of caspase-3 was also detected. Our results indicate that caspase-3, caspase-6 and genes coding Bcl2 family proteins play a key role in the apoptotic response in *Lagovirus europaeus*/GI.2 infection in organs that are not the target of virus replication.

## 1 Introduction

*Lagovirus europaeus* is a virus that causes a severe disease in rabbits (*Oryctolagus cuniculus*) called Rabbit Hemorrhagic Disease (RHD) (Abrantes et al., 2012) and it belongs to the genus Lagovirus of the *Caliciviridae* family (Vinjé et al., 2019). Within *Lagovirus europaeus* associated with RHD, genotypes and variants have been distinguished. *Lagovirus europaeus*/GI.1 was first detected in domestic rabbits in China in 1984 (Liu et al., 1984) and has been occurring worldwide since then. Subsequently, in 1997 the first antigenic variant of this virus appeared in Italy and it forms the GI.1a group (Capucci et al., 1998). The presence of a second genotype of the virus has been confirmed relatively recently, as *Lagovirus europaeus*/GI.2 was detected in France in 2010 (Le Gall-Reculé et al., 2011) with particular characteristics such as infecting young and adult animals, but also inducing RHD not only in rabbits, but also in other Lagomorpha species (Bębnowska and Niedźwiedzka-Rystwej, 2019).

Research involving *Lagovirus europaeus* infection in rabbits is an important direction and is successfully used in dealing the problem of acute liver failure, particularly of viral origin (Tunon et al., 2003). In addition, RHD has several similarities to human viral hemorrhagic fever (VHF). *Lagovirus europaeus*/GI.1 and GI.2 studies can be used as a research model for VHF, which are still a serious problem in the world (Bębnowska and Niedźwiedzka-Rystwej, 2022). *Lagovirus europaeus*/GI.1 and GI.2 infection can occur in three different clinical forms, which include acute, peracute, and chronic. In the peracute course, the sudden death of an infected animal is observed, in which only weakly expressed disease symptoms, or their complete absence have been diagnosed. In the acute form, the symptoms are strong and clear, which is also observed in the chronic course, in which survival is significantly higher, and the history of the disease determines the occurrence of natural protection against re-infection (Abrantes et al., 2012). Animal death occurs as a consequence of the development of disseminated intravascular coagulation as a result of progressive viral replication, thereby leading to multiple organ failure.

Apoptosis is responsible for the physiological removal of unwanted cells, such as damaged or aging cells, in mature tissues. Activation of this process can be induced in the cell by the external pathway through death receptors and by the internal mitochondrial pathway (Thomson, 2001). The external apoptotic pathway begins with the stimulation of death receptors after binding their ligands. As a result of this stimulation, the recruitment of the Fas-associated death domain (FADD) and caspase-8 occurs, which then activates the effector caspases-3, and − 7, leading to apoptosis (Elmore, 2007). Executive caspases also include caspase-6, but in apoptosis it is often not activated by initiator caspases, but by caspase-3 (Wang et al., 2015). The internal pathway is activated as a result of intracellular apoptotic signals, and its modulator is the BH3 proteins. The pro-apoptotic Bax and Bak proteins located on the mitochondrial outer membrane (MOM) are then activated (Elmore, 2007). Permeabilization of MSM releases i.a. cytochrome c, which activates caspase-9 - an activator of effector caspases-3 and -7 leading to apoptosis. Moreover, the cleavage of poly (ADP-ribose) polymerase (PARP protein) is a factor that stimulates apoptosis through the internal pathway (Los et al., 2002). The p53 protein also takes part in the regulation of apoptosis, and influences the expression of pro-apoptotic and anti-apoptotic proteins and many components involved in the apoptotic pathway (Elmore, 2007).

Programmed cell death plays one of the key roles in the pathogenesis of RHD and affects a variety of cells. Given that hepatocytes are the target site of viral replication, most of the available studies focus on the analysis of this organ. We have previously conducted more detailed studies of apoptosis in immune cells using nine *Lagovirus europaeus*/GI.1 strains. We showed that apoptosis is expressed by changes in the percentage of apoptotic granulocytes and lymphocytes (Niedźwiedzka-Rystwej and Deptuła, 2012; Niedźwiedzka-Rystwej et al., 2013). However, the presence of apoptotic cells in *Lagovirus europaeus*/GI.1 infection has been confirmed in abdominal macrophages, epithelial cells, lungs, kidneys, heart, spleen and lymph nodes (Alonso et al., 1998). Furthermore, several studies show that during infection with GI.1a strains, a different immune picture is observed than in other genotypes within the GI.1 group, and these differences mainly concern cell-specific and humoral immunity and the process of apoptosis (Niedźwiedzka-Rystwej and Deptuła, 2012; Niedźwiedzka-Rystwej et al., 2013). These studies indicate that the biological features of *Lagovirus europaeus*/GI.1 strains (hemagglutination capacity, antigenic variability) influence the immune response (Niedźwiedzka-Rystwej and Deptuła, 2012, 2023; Niedźwiedzka-Rystwej et al., 2013).

To date, there have been no widely studies analysing apoptosis activation in GI.2 strain infection (Neimanis et al., 2018). The current study aimed to evaluate the activation of the apoptotic cell death in the selected organs in Rabbit Haemorrhagic Disease caused by *Lagovirus europaeus*/GI.2 in order to better understanding the pathogenesis of the disease caused by the GI.2 genotype strain.

## 2 Materials and methods

### 2.1 Experimental model

The experimental infection of animals was carried out at the Pomeranian Medical University in Szczecin based on the consent obtained by the Local Ethical Committee for Experiments on Animals in Poznań No. 35/2022.

The research was carried out on European rabbits *Oryctolagus cuniculus*/Crl:KBL (NZW)/0052 not vaccinated against *Lagovirus europaeus*/GI.1 and GI.2 purchased from a licensed breeder (AnimaLab Sp. z o.o., Poznań, Poland). The experiment included 20 animals (6 weeks old) weighing in the range 4,5 kg (±10%): the control group (*n* = 10) and the study group infected with the GI.2 strain (*n* = 10). In each group, 50% were males and 50% females. After being transported to the laboratory, rabbits were allowed a three-week adaptation period. The animals were provided with complete feed and had unlimited access to water.

The virus used in the study (RHDV2_Ri2017 strain; GenBank: OQ680671) came from rabbits that died from a naturally acquired infection in Italy. The inoculum was prepared from liver sample according to the method described earlier (Niedźwiedzka-Rystwej and Deptuła, 2023). Infection was provoked by the intramuscular injection of 2 × 10^4^ haemagglutination units of the virus (Tunon et al., 2003; Tuñón et al., 2011; Laliena et al., 2012), which was determined by the haemagglutination assay. The haemagglutination assay was performed using the plate method. The study used a 0.75% solution of type 0 human red blood cells in phosphate-buffered saline (PBS). Then, two-fold serial dilutions of the previously prepared purified liver homogenate were prepared. The same amount of blood cell solution was added to the wells containing 0.1 mL of the next dilution of the homogenate. PBS was used as a negative control. The plates were then incubated for 60 min at room temperature. The hemagglutination titer was obtained at a 1:16 dilution. The infectious titer of the virus inoculum (1 mL) was determined to 1,250 u/mL [assuming that 1 HA unit corresponds to 10^4^ particles per ml (Ryu, 2017)].

The time of inoculum administration was marked as 0 h of the experiment. Rabbits in the control group were given placebo (1 mL) in the same way in the form of PBS (phosphate-buffered saline). After inoculation, the health of all rabbits was monitored at least twice a day and the body temperature was measured in anus twice a day. The onset of severe symptoms of Rabbit Haemorrhagic Disease was considered the terminal moment of the experiment. Animals qualified for euthanasia were anesthetized by intravenous administration of the preparation sodium pentobarbital at 90 mg/kg, followed by administration of the cardiac arrest-inducing preparation sodium pentobarbital at 250 mg/kg. Tissue samples were collected post-mortem and were placed in tubes containing RNAlater RNA Stabilization Solution (Thermo Fisher Scientific, Waltham, Massachusetts, United States). All the samples were stored at −80°C until analysis.

### 2.2 Real-time PCR

Total RNA was isolated from frozen tissue samples using RNeasy Mini Kit (A&A Biotechnology, Poland) following the manufacturer’s instructions. The quantity and quality of the isolate was determined using a NanoDrop ™ 2000 spectrophotometer (Thermo Fisher Scientific, Waltham, Massachusetts, United States). The integrity of the purified RNA samples was tested by 1% (w/v) agarose gel electrophoresis. Total RNA was reverse transcribed using the RevertAid First Strand cDNA Synthesis Kit (Thermo Fisher Scientific, Waltham, Massachusetts, United States) as recommended by the manufacturer. Relative expression of selected genes was assessed by real-time PCR using the LightCycler® 480 Instrument II (Roche Diagnostics GmbH, Mannheim, Germany) and PowerUp™ SYBR™ Green Master Mix Thermo Fisher Scientific, (Waltham, Massachusetts, United States) was used according to manufacturer’s recommendations. In the study specific primers were used (Table 1). Threshold cycle (Ct) values obtained by real-time PCR analysis were normalized by *β-actin* gene expression, and the relative expression of individual genes was calculated using the Pfaffl method (Pfaffl, 2001).

**Table 1:** Primers used in the study (Sudo and Minami, 2011; Chen et al., 2019).

Gen	Forward primer (5′-3 ′)	Reverse primer (5′-3 ′)
*Caspase-3*	GCTGGACAGTGGCATCGAGA	TCCGAATTTCGCCAGGAATAGTAA
*Bax*	GTGTCTCAAGCGCATTGGCG	CAAACATGTCGGCCTGCCACT
*Bcl2*	GATTGTGGCCTTCTTTGAGTTC	AAGTCTTCAGAGACACCCAGGA
*β-actin*	TGGCATCCTGACGCTCAA	TCGTCCCAGTTGGTCACGAT

### 2.3 Western blot analysis

Levels of selected protein were estimated using extracts from lungs, hearts, kidneys and spleens collected from rabbits treated with T-PER™ Tissue Protein Extraction Reagent (cat.no. 78510, Thermo Scientific™) and Bead Ruptor Elite (cat.no. SKU 19-040E, Omni International). The relative levels of selected proteins were determined by Western blot experiments using a WES system (WES — Automated Western blots with Simple Western; ProteinSimple, San Jose, CA, United States). Specific proteins were detected with the following antibodies: Cleaved Caspase-3 (Asp175) (5A1E) Rabbit mAb (#9664, Cell Signaling Technology), Cleaved Caspase-6 (Asp162) Rabbit mAb (#9761, Cell Signaling Technology) and Cleaved PARP (Asp214) (D64E10) Rabbit mAb (#5625, Cell Signaling Technology).

For the detection procedure, secondary antibodies (anti-rabbit), which were included in an anti-rabbit detection module (#DM001 ProteinSimple, San Jose, CA, United States) were used. The total protein level, determined using a total protein detection module for chemiluminescence (#DM-TP01, ProteinSimple, San Jose, CA, USA), was used as the loading control. Quantification of the results was performed using the software included in the WES system.

### 2.4 Caspase-3 activity

Caspase-3 activity assay was performed using the Caspase-3 Activity Assay Kit (#5723, Cell Signaling Technology) according to the manufacturer’s recommendations. Samples with a protein content of 0.5 mg were prepared for the experiment. Fluorescence measurement was performed on the EnSpire (PerkinElmer, Waltham, Massachusetts, USA) device using excitation at 380 nm and emission at 440 nm wavelength, with gain value 60.

### 2.5 Statistical analysis

The statistical analysis was performed with Tibco Statistica 13.3 (StatSoft, Palo Alto, CA, USA). Normal distribution of continuous variables was tested using Shapiro–Wilk test. In order to compare the 2 groups, data with a normal distribution were analysed using the Student’s t-test. The Mann–Whitney U test was used to analyse data with a non-normal distribution. All values are expressed as the mean ± standard error (SE) or standard deviation (SD).

## 3 Results

### 3.1 Clinical signs of disease and post-mortem analysis

**Figure 1:**
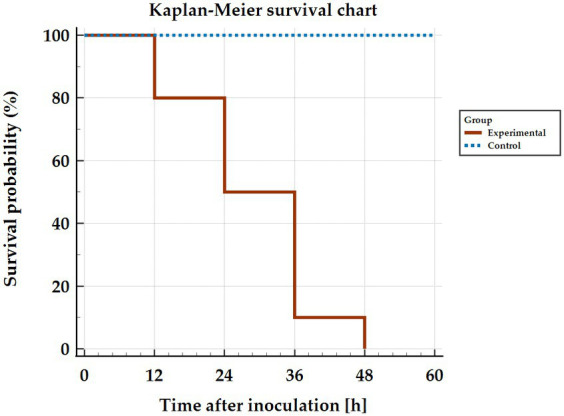
Kaplan–Meier curve showing survival of rabbits infected with *Lagovirus europaeus*/GI.2.

Observation of signs during infection showed that in some animals (n = 6) no clinical signs were registered and death occurred suddenly at 12–36 h p.i. Figure 1 shows the survival curve of infected rabbits. The remaining animals (*n* = 4) developed an acute form of the disease and clinical signs (apathy, conjunctival congestion, dyspnoea, body temperature > 41°C, anorexia) were observed from 24 h p.i. onwards. The experimental endpoints for these animals were reached at 36–48 h p.i. The key symptoms of the disease qualify for euthanasia included: neurological symptoms, shortness of breath, nosebleeds. Post-mortem analysis of animals in the infected group showed the presence of typical RHD features in all rabbits.

### 3.2 *Lagovirus europaeus*/GI.2 infection promotes *caspase-3* gene expression

**Figure 2:**
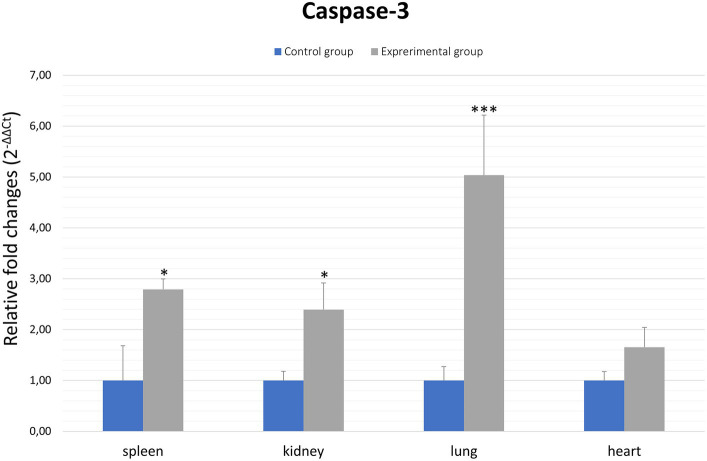
Expression levels of *caspase-3* mRNA in selected organs in rabbits infected with *Lagovirus europaeus*/GI.2. Real-time PCR results showed that expression level of *caspase-3* mRNA was higher in experimental group than in control group. All values are presented as the mean ± standard error (SE). ^*^p ≤ 0.05; ^**^p ≤ 0.03; ^***^p ≤ 0.01.

Caspases are a family of cysteine proteases that are the executors of apoptosis. The caspases involved in the apoptotic cell death process are divided into two groups: initiator and effector caspases. Initiator caspases are responsible for the activation of effector caspases, which include caspase-3. Caspase-3 is involved in both death receptor-mediated apoptosis, but also via the mitochondrial pathway, therefore this protein is an important element of apoptotic flux. Thus, to assess apoptosis activity in organs (spleen, kidney, lung and heart) of rabbits infected with *Lagovirus europaeus*/GI.2 we decided to assess the expression of the *caspase-3* gene. The results show that there is a statistically significant differences between experimental and control groups in the lungs (*p* ≤ 0.01), spleen (p ≤ 0.01) and kidney (*p* ≤ 0.03). No statistically significant differences were observed in comparing results from heart tissue (Figure 2; Table 2).

**Table 2:** Results of apoptotic markers determined by real-time PCR.

Gen	Parameter	Organ							
		Spleen		Kidney		Lung		Heart	
*Caspase-3*	Group	CG	EG	CG	EG	CG	EG	CG	EG
	Mean	1.00	3.30	0.98	3.32	1.00	4.37	1.00	1.31
	SD	0.60	1.99	0.58	2.16	0.96	2.55	0.83	0.57
	SE	0.19	0.63	0.18	0.68	0.30	0.81	0.26	0.18
	*p*-value	0.007***		0.017**		0.007***		0.791	
*Bax*	Mean	1.01	1.50	1.00	2.92	1.00	2.64	0.99	1.32
	SD	0.39	0.50	0.60	1.14	0.43	1.91	0.62	0.94
	SE	0.12	0.16	0.19	0.36	0.13	0.61	0.20	0.30
	*p*-value	0.037*		0.001***		0.075		0.969	
*Bcl2*	Mean	1.00	0.36	1.00	0.26	0.98	1.69	0.99	0.77
	SD	0.39	0.27	0.89	0.31	0.96	1.67	0.85	0.46
	SE	0.12	0.09	0.28	0.10	0.30	0.53	0.27	0.15
	*p*-value	0.004***		0.001***		0.075		0.273	
*Bax/Bcl2* ratio	Mean	0.99	4.90	1.02	15.89	1.02	4.00	1.01	2.74
	SD	0.53	3.26	0.48	15.26	1.22	4.21	1.45	3.26
	SE	0.17	1.01	0.15	4.82	0.29	2.43	0.46	1.03
	*p*-value	0.03**		0.0001***		0.069		0.275	

SD-standard deviation; SE-standard error; ^*^p ≤ 0.05; ^**^p ≤ 0.03; ^***^p ≤ 0.01.

### 3.3 Relative expression of *Bcl2* and *Bax* mRNA in selected organs during *Lagovirus eurpaeus*/GI.2 infection

**Figure 3:**
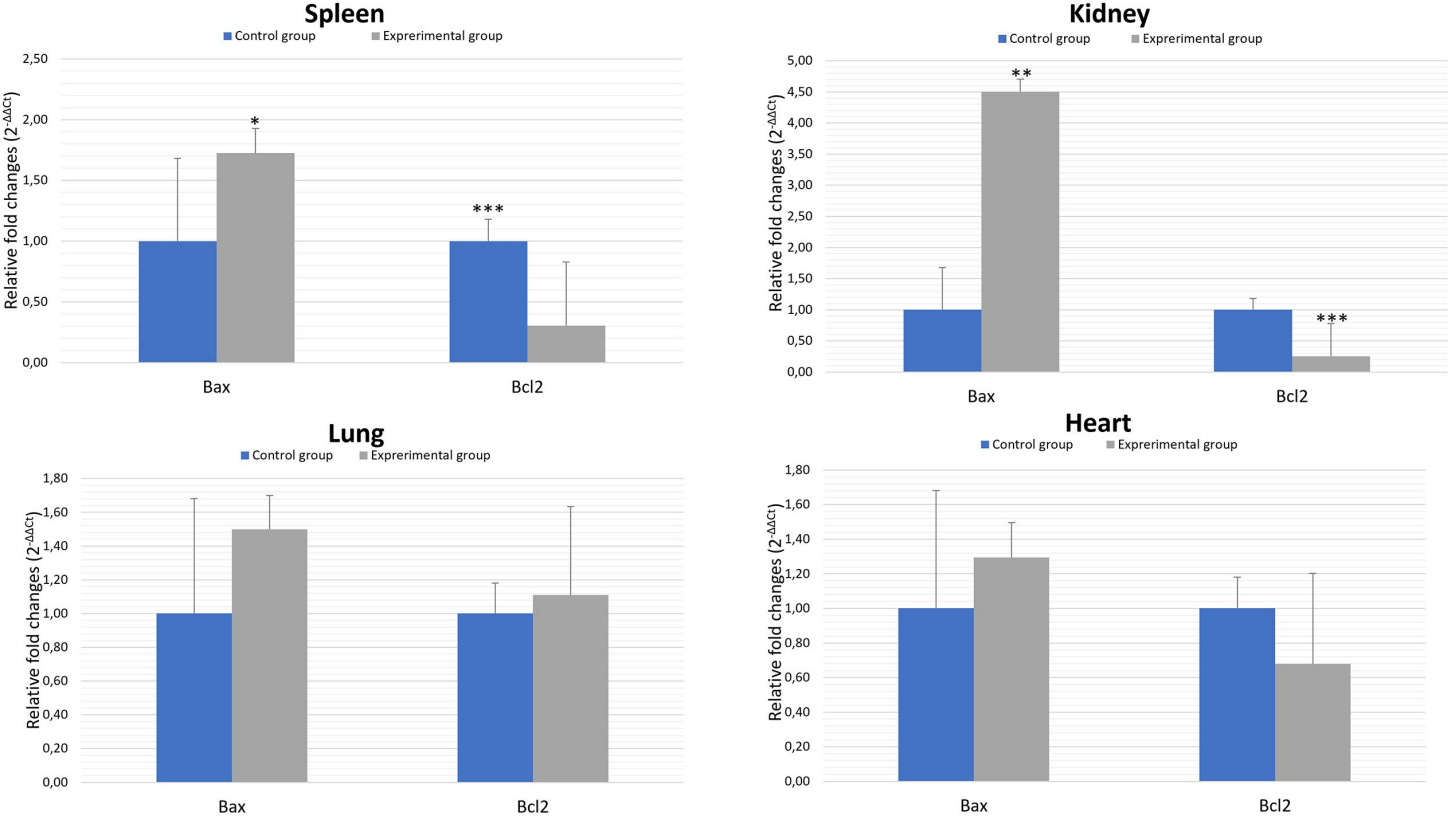
Expression levels of *Bax* and *Bcl2* mRNA in selected organs in rabbits infected with *Lagovirus europaeus*/GI.2. All values are presented as the mean ± standard error (SE). ^*^p ≤ 0.05; ^**^p ≤ 0.03; ^***^p ≤ 0.01.

Bcl-2 family proteins belong to the regulators of apoptosis and are distinguished between proapoptotic (Bax) and anti-apoptotic (Bcl2) proteins. Our results showed statistically significant differences between experimental and the control group in the gene expression of proapoptotic *Bax* in the kidney (*p* ≤ 0.01) and spleen (*p* ≤ 0.05). Analysis of the results of the anti-apoptotic *Bcl2* gene expression showed a statistically significant differences between experimental and control groups in kidney and spleen (*p* ≤ 0.01). There were no significant differences between experimental and control groups in the *Bcl2* gene expression in the lung and heart tissues (Figure 3; Table 2).

**Figure 4:**
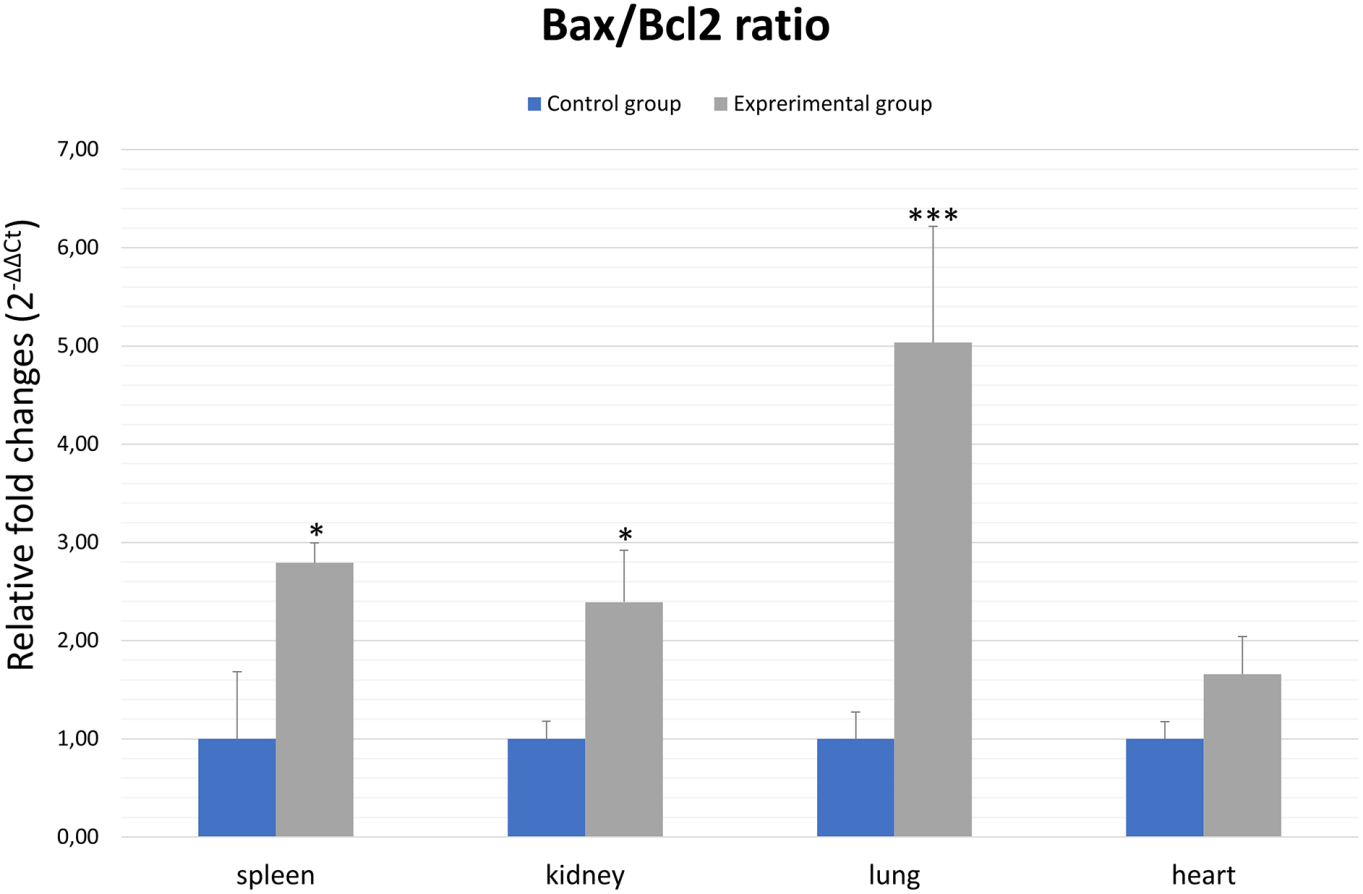
*Bax/Bcl2* ratio in selected organs in rabbits infected with *Lagovirus europaeus*/GI.2. All values are presented as the mean ± standard error (SE). ^*^p ≤ 0.05; ^***^p ≤ 0.01.

In general, the ratio of proapoptotic to anti-apoptotic protein gene expression indicates the course or inhibition of apoptotic cell death. For this reason, we also analysed the *Bax*/*Bcl2* mRNA expression ratio. Our results indicate that there was an elevated *Bax/Bcl2* mRNA expression ratio in the organs of infected rabbits compared to the control group in the kidney and spleen (*p* ≤ 0.01). In the lungs and heart there was no significant changes (Figure 4; Table 2).

### 3.4 Relative levels of cleaved caspase-3, caspase-6 and PARP and activity of caspase-3 in selected organs of infected rabbits

**Figure 5:**
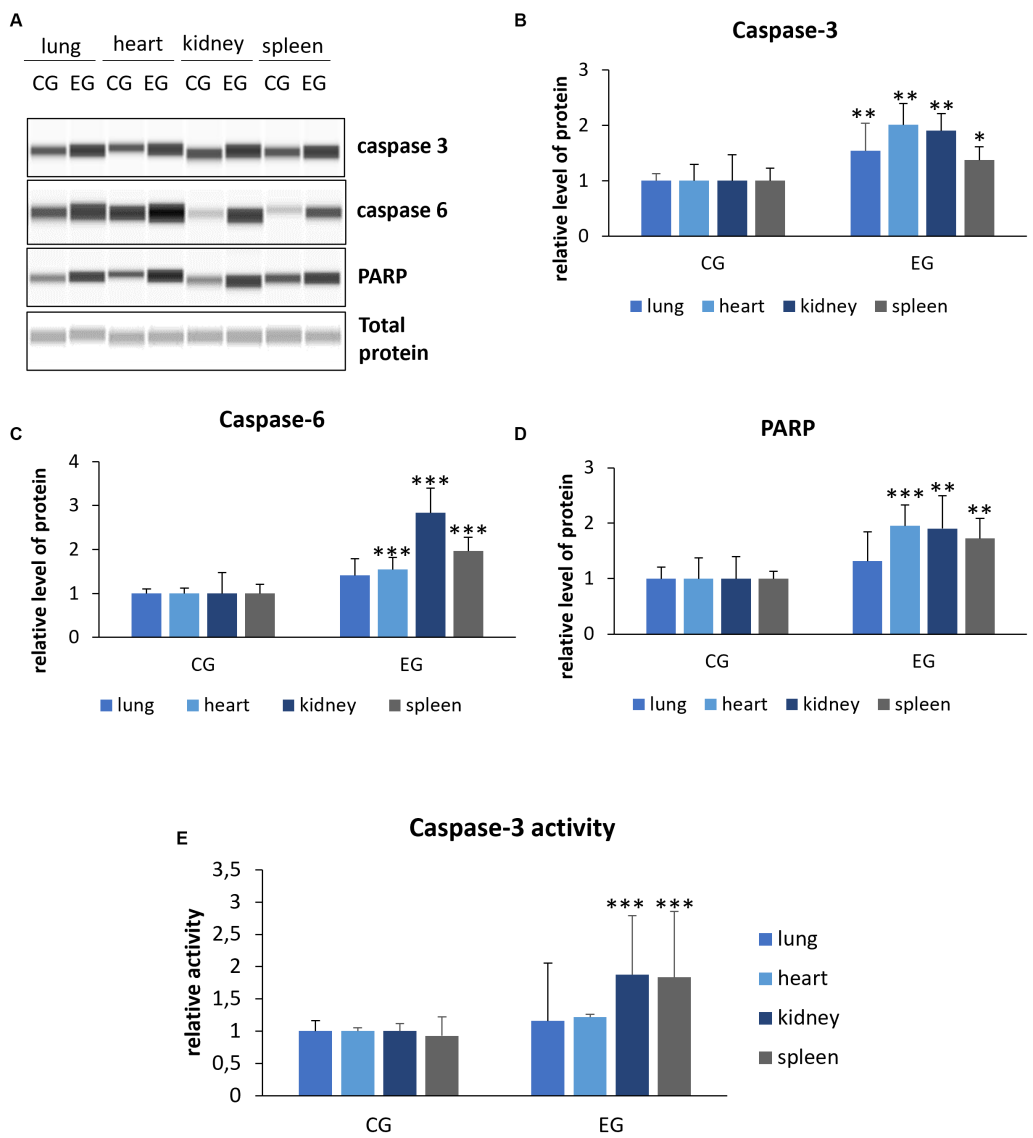
Relative levels of cleaved proteins **(A–D)** and caspase-3 activity **(E)** in selected organs. Protein levels were assessed using Western blot method (the WES system). Panel **(A)** shows representative blots of cleaved proteins related to apoptosis. In panels **(B–D)**, there are indicated levels of cleaved caspase-3, caspase-6 and PARP. Panel **(E)** indicates caspase-3 activity determined by colorimetric assay. All values are expressed as mean value ± standard deviation (SD). ^*^p ≤ 0.05; ^**^p ≤ 0.03; ^***^p ≤ 0.01. CG-control group; EG-experimental group.

Activation of effector caspases, which include caspase-3 and caspase-6, occurs through cleavage of specific internal Asp residues that separate the large and small subunits (Shi, 2002). Thus, to gain more insight into the mechanism of apoptotic cell death, we also determined the levels of cleaved caspase-3 and caspase-6. The results showed elevated levels of caspase-3 compared to controls in all organs: lung, heart, kidney (*p* ≤ 0.03) and spleen (*p* ≤ 0.05). Analysis of caspase-6 showed increased levels in the heart, kidney and spleen (*p* ≤ 0.01) in the infected rabbits compared to the control group, while no significant changes were observed in the lungs (Figure 5). In our study, we also determined the level of PARP protein, whose cleavage leads through an intrinsic pathway to the activation of apoptosis. Our results indicate elevated levels of the cleaved form of PARP in the heart (*p* ≤ 0.01), kidney and spleen (*p* ≤ 0.03). No significant changes were observed in the lungs (Figure 5).

Given that caspase-3 is a key executor of apoptosis, we also decided to determine its activity. We registered that caspase-3 activity is increased in the kidney and spleen (*p* ≤ 0.01) of rabbits from the experimental group compared to the control, while no significant difference was observed in the lung.

## 4 Discussion

Apoptosis is the best-understood type of programmed cell death and is an important component of the cellular response to damage. The particular role of apoptosis is highlighted in infectious states, as in viral infections many cells enter the apoptotic pathway to limit the production and release of progeny virions. Nevertheless, viruses have developed specific mechanisms that enable them to modulate apoptosis in host cells (Thomson, 2001). Dengue virus has been shown to induce apoptosis in many cell types, suggesting that this process may play an important element in the pathogenesis of the disease (Martins et al., 2012). Strong intravascular apoptosis, including leukocyte apoptosis, has also been demonstrated in Ebola virus infection, where it has been shown to correlate with the risk of patient death (Baize et al., 1999). Increased rates of apoptosis have also been demonstrated in Crimean Congo Haemorrhagic Fever infection, where autophagic lesions have been demonstrated in peripheral blood leukocytes, and the expression of genes related to autophagy has also been demonstrated (Guler et al., 2016).

The first reports of apoptosis in calicivirus infection came from Alonso et al. (1998), who observed hallmarks of apoptosis in hepatocytes in rabbits infected with *Lagovirus europaeus*, indicating that this process is involved in the pathogenesis of the disease. Given that apoptosis in Rabbit Haemorrhagic Disease involves cells that sustain viral replication, such as pulmonary macrophages, intravascular monocytes, endothelial cells (Alonso et al., 1998), as well as granulocytes and lymphocytes (Niedźwiedzka-Rystwej and Deptuła, 2012; Niedźwiedzka-Rystwej et al., 2013), it is hypothesized that activation of this process may be related to the viral strategy to spread infection in the host. This theory is also supported by studies conducted with substances showing hepatoprotective effects in *Lagovirus europaeus* infection such as melatonin (Tuñón et al., 2011), cardiotrophin-1 (Tunon et al., 2011) and N-acetylcysteine (San-Miguel et al., 2006). The protective effect of these substances is presumed to be related to the activation of anti-apoptotic factors.

Our study showed increase in *caspase-3* gene expression in *Lagovirus europaeus*/GI.2-infected rabbits in spleen, kidney and lung tissue in compare to the control group. We have also detected elevated levels of caspase-3 in all organs, but activity of this protein was not significant only in lung tissue. Moreover, higher levels of caspase-6 were observed in heart, kidney and spleen tissue compared to the control group. Caspases are among the key mediators of apoptotic cell death. Caspase-3 activation pathways may be dependent or independent of cytochrome c release from mitochondria and caspase-9 function. The role of caspase-3 in apoptosis is crucial, as it is required for apoptotic chromatin condensation and DNA fragmentation (Porter and Jänicke, 1999). Moreover, cleavage of PARP during apoptotic cell death is performed by caspase-3 (Los et al., 2002). In this study we have also detected elevated levels of cleavage PARP in heart, kidney, and spleen of infected rabbits compared to the control group.

Admittedly, in this study we registered some discrepancies in the observation of gene expression levels determined by real-time PCR and the relative number of proteins determined by Western blot method. Also, the protein levels did not always match the protein activity. However, it should be remembered that the determination of mRNA levels gives insight into the initial phase of the chain of regulatory events, and moreover, protein abundance and activity is the result of regulation by many complex mechanisms. Another important aspect is also that mRNA and proteins have different rates of synthesis and degradation. Similarly, the level of gene expression does not clearly indicate the amount of end product formed, but in general, more mRNA is associated with more protein. With these facts in mind, a measure of forbearance should be exercised during interpretation of the results (Payne, 2015).

García-Lastra et al. (2010) showed that in rabbits infected with RHD virus, caspase-3 activity was noticeable in the liver 36 h (12.5-fold of control) and 48 h (12.6-fold) after inoculation. Furthermore, Chen et al. (2019) showed, that ectopic expression of the gene coding non-structural protein 6 (NSP6) of RHD virus led to increased activation of caspase-3, but also −8 and − 9 in kidney cell line from rabbit (RK13 cell). These results indicate that effector caspase-3-mediated activation of apoptosis occurs in *Lagovirus europaeus* infection. Induction of apoptosis by another calicivirus was confirmed by Sosnovtsev et al. (2003). The authors showed that infection of feline Crandell-Rees kidney cells (CRFK) by feline calicivirus (FCV) caused induction of changes typical of apoptosis such as translocation of phosphatidylserine to the outer membrane of the cell, chromatin condensation and fragmentation of oligonucleosomal DNA. Moreover, an increase in caspase-3, −8 and − 9 activity was also recorded, with caspase-3 activity being the most strongly expressed.

In our study, we also analyzed the expression of genes coding apoptotic regulators from the Bcl-2 family. Proteins from this family can exhibit anti-apoptotic or pro-apoptotic effects. In general, the ratio between these proteins in the mitochondria determines cell death or survival. Thus, overproduction of anti-apoptotic proteins i.a. Bcl-2 protects the cell from apoptosis, whereas a predominance of pro-apoptotic proteins i.a. Bax leads to the induction of apoptotic cell death through the formation of transition pores allowing the release of cytochrome c. In a study by San-Miguel et al. (2006), apoptosis induced in *Lagovirus europaeus*/GI.1 infection was associated with modulation of *Bcl-2* and *Bax* genes. As a result, the relative expression of *Bax* to *Bcl-2* was higher at 36 and 48 h after infection in the infected rabbits compared to healthy control. Similar results were also obtained by Chen et al. (2019), where *Bax* expression was elevated in NSP6-transfected cells at 24 and 36 h post-transfection compared to the vector control. In contrast, *Bcl-2* expression was lower in NSP6-transfected cells at 12 and 36 h post-transfection compared to the vector control. Furthermore, NSP6-transfected cells showed an increased ratio of *Bax* to *Bcl-2* indicating that NSP6-induced apoptosis involves members of the Bcl-2 family. Our results show that there were a significant differences between experimental and control groups in the relative expression of *Bax* to *Bcl-2* in spleen and kidney cells. Admittedly, there were no differences between analyzed groups in *Bax*/*Bcl2* mRNA values in the lungs and heart. Given that our results showed a significant differences between comparing groups in *caspase-3* gene expression in the lung (*p* ≤ 0.01), it would be important to further investigate whether there was a strong stimulation of expression of the genes involved in external apoptotic pathway in this case. This is important because San Miquel et al. recorded that in *Lagovirus europaeus*/GI.1 infection occures increased *FasL* gene expression at 36 and 48 h.p.i (San-Miguel et al., 2006).

## 5 Conclusion

Our findings indicate for the first time that in *Lagovirus europaeus*/GI.2 infection there is an increased expression of apoptotic genes and elevated levels of key proteins in organs that are not conventionally considered to be the target site of viral replication. The analysis of obtained results allows us to conclude that the role of caspase-3 and caspase-6 for this process seems to be crucial. Our research undoubtedly contributes important data to a better understanding of Rabbit Haemorrhagic Disease caused by GI.2 strain. However, this work has some limitations as we only analyzed a few selected markers of apoptosis. In particular, it seems important to extend research into markers of the external apoptotic pathway. Another limitation of this study is that whole organs were analysed without specifying individual cell types. Due to these facts further studies are required to better understand the role of apoptosis in the pathogenesis of RHD caused by *Lagovirus europaeus*/GI.2 infection and the involvement of individual components of the apoptotic reaction cascade.

## Data availability statement

The original contributions presented in the study are included in the article/supplementary materials, further inquiries can be directed to the corresponding author/s.

## Ethics statement

The animal study was approved by Local Ethics Committee in Poznań The University of Life Sciences in Poznań Wołyńska 35, 60-637 Poznań. The study was conducted in accordance with the local legislation and institutional requirements.

## Author contributions

DB: Conceptualization, Data curation, Formal analysis, Investigation, Methodology, Project administration, Resources, Software, Validation, Visualization, Writing – original draft. RH: Data curation, Investigation, Software, Visualization, Writing – review & editing. KW: Investigation, Visualisation, Writing-review & editing. MZ: Investigation, Visualisation, Writing – review & editing. ER: Investigation, Visualisation, Writing – review & editing. KP: Investigation, Methodology, Writing – review & editing. PN-R: Funding acquisition, Project administration, Supervision, Validation, Writing – review & editing.
